# They are what you eat: Shaping of viral populations through nutrition and consequences for virulence

**DOI:** 10.1371/journal.ppat.1008711

**Published:** 2020-08-13

**Authors:** Rebekah Honce, Stacey Schultz-Cherry

**Affiliations:** 1 Department of Infectious Diseases, St. Jude Children’s Research Hospital, Memphis, Tennessee, United States of America; 2 Integrated Program in Biomedical Sciences, Department of Microbiology, Immunology and Biochemistry, University of Tennessee Health Science Center, Memphis, Tennessee, United States of America; University of Michigan Medical School, UNITED STATES

## Introduction

**Fig 1:**
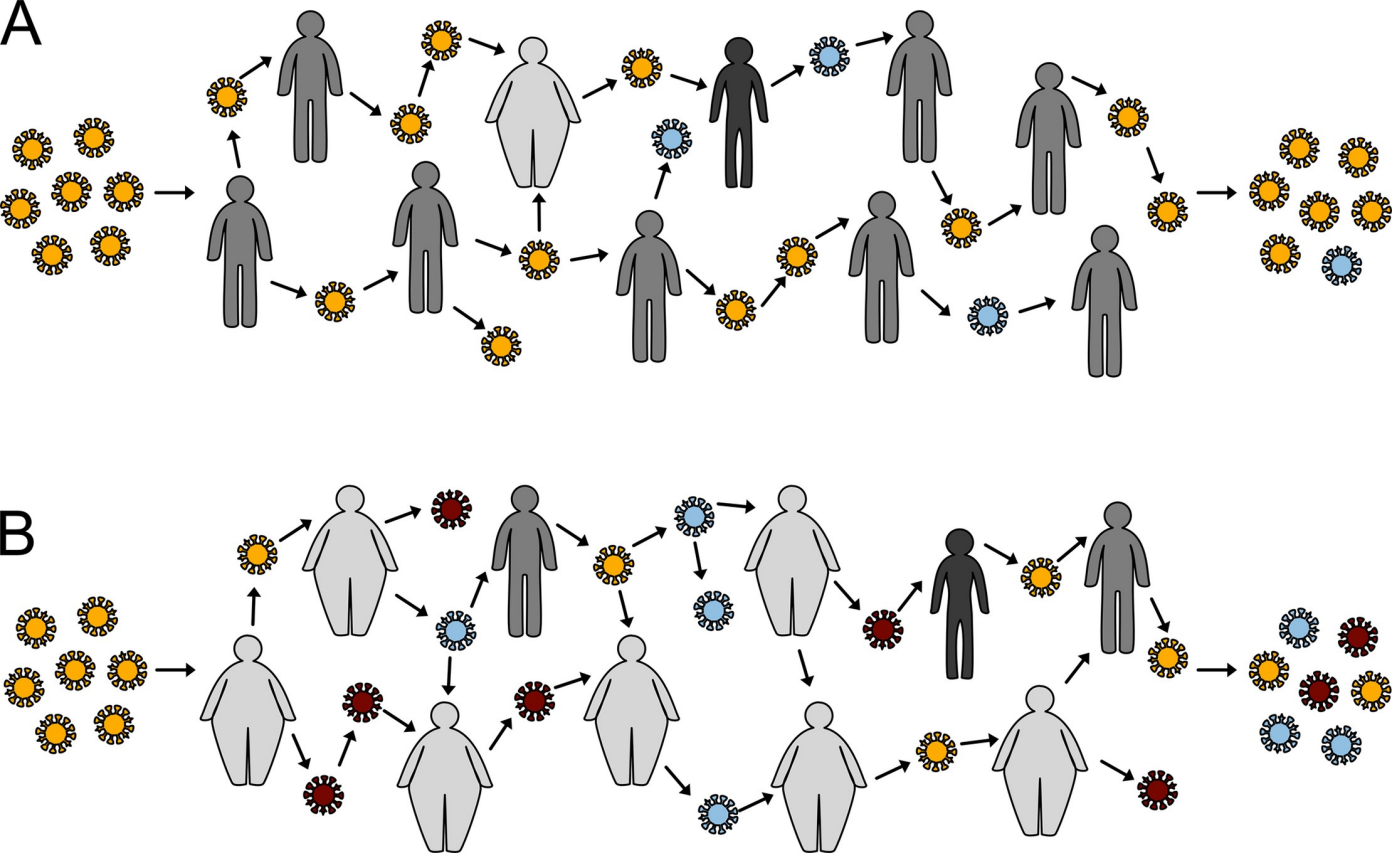
Prevalence of malnutrition may impact global viral evolutionary patterns. Worldwide rates of obesity are projected to reach over 50% by 2050. Additionally, undernutrition of both children and adults continues to be a global health crisis. (A) In a primarily healthy, lean environment, viral spread and acquisition of variants is limited as a result of robust immune responses. (B) As the incidence of malnourishment has increased, we have discovered that the resulting diminished immunity can permit the acquisition of minor variants and a virulent phenotype. In our current environment of widespread obesity, global viral evolutionary patterns may change because of increased host susceptibility and decreased host immunity.

Humans have coexisted with viral pathogens for tens of thousands of years, influencing both their emergence and evolution. However, the pervasiveness of the Western diet and disparities in food access and security have altered how we as hosts interact with our viral pathogens. Malnutrition, the state of having insufficient, excess, or imbalanced sources of energy, is well known to attenuate immune responses. Could nutrition also actively shape how viruses evolve? Malnourishment is a global, intersectional issue, and it may soon force a revision of our understanding of how viruses evolve within their hosts (**Fig 1**) [1].

## Why do RNA viruses form quasispecies?

First theorized over 4 decades ago [2], a quasispecies population structure has been documented in plant, animal, and human pathogens [3–5]. A viral quasispecies describes the mutant but related genomes that collectively infect, replicate, and spread among hosts. Traditionally, the theory has been applied to RNA viruses. Because of their short generation times, small genomes, and the inherent lack of proofreading in most RNA replication, single nucleotide variants (SNVs) emerge at a rate of roughly 10^3^ to 10^7^ more mutations per nucleotide copied compared with DNA viruses [6].

Nonsynonymous SNVs are continuously accrued and purged from the viral genome. This flux generates a related “swarm” of viruses, which have little effect on the consensus sequence but may show phenotypic differences. Mutations with phenotypic consequences are generally deleterious; very few mutations have any fitness benefit. However, if beneficial mutations arise, they may relate to host range, drug resistance or vaccine escape, and replicative capacity [7, 8]. Both beneficial and the common deleterious mutations balance the structure of the viral swarm through complementation, interference, and cooperation [9–11]. Within a single host, tissue-specific subpopulations may vary in virulence without affecting consensus sequence or phenotype [12, 13]. Importantly, the consensus sequence should not be considered the “fittest sequence,” because selection, competition, and genetic drift act upon the entire viral swarm. Therefore, fitness of the swarm exceeds clonal sequence fitness, highlighted by work in vesicular stomatitis virus [3] and bacteriophage systems [14].

Viruses are obligate intracellular parasites that require a host cell to complete their life cycle. Barriers to replication exist within and between susceptible hosts, which restrict viral population diversity to quell infections [13]. In these wide-ranging environments, a heterogenous viral swarm containing isolates with differing abilities to infect, transmit, and survive environmental and immunological onslaughts may safeguard viral existence. However, this genetic plasticity has bounds, with an evolutionarily beneficial middle ground between high- and low-fidelity replication [15, 16]. The “Goldilocks” approach maximizes fitness by avoiding lethal mutagenesis while ensuring amenability to selective pressures [17]. Too low fidelity leads to error catastrophe and collapse of the viral population; conversely, a highly clonal population may be extinguished by host defenses [18–21].

## What is the implication of viral diversity on disease severity?

Numerous theories have questioned the biological relevance of a quasispecies and challenged its significance [17, 22]. However, boosting genetic diversity—to a point—is theorized to increase virulence. A viral swarm may be better equipped to face bottlenecks imposed by infecting hosts, environmental persistence, and transmission. Even within a single host, blockades due to infection barriers and the immune response diminish sequence variation, leaving a relatively homogenous population until replicative errors replenish the mutant pool [13]. So, do viruses harboring higher genetic diversity initially fare better in establishing an infection and displaying virulent phenotypes?

In studies with classical swine fever virus, higher genetic diversity correlated with virulence [23]; however, this conclusion has been challenged [24]. In other animal viruses, diversity increases precede the selection of virulent genomes [4]. Parallel conclusions have been made for human pathogens. In hepatitis C virus (HCV)-positive patients, high viral diversity prior to transplantation correlated with higher liver fibrotic scoring 1 year post-transplantation [5]. Continued genetic evolution of HCV correlated with progressing hepatitis, whereas resolution was associated with genetic stasis of HCV population [25, 26]. A model low-fidelity RNA-dependent RNA polymerase (RdRp) poliovirus variant demonstrates that increasing genetic diversity may not always yield fit populations [10, 27], yet high-fidelity RdRp mutants producing nearly clonal populations display reduced fitness in vivo [21].

## Do host characteristics influence quasispecies structure?

Selection pressures ranging from host antiviral responses to pharmaceutical interventions mold the viral swarm. Upon infection, immune responses restrict genetic diversity by limiting spread and replication, eloquently demonstrated using a model poliovirus RdRp [13, 20]. Host immunological status is implicated in molding the quasispecies of dengue virus [28], norovirus [29], influenza virus [30], and coronavirus [31], among others. From these findings, empirical studies have found that host features responsible for attenuating immunity are also implicated in shaping the quasispecies and virulence, including aging [32] and immunocompromised status [29, 30, 33, 34].

Exogenous control of infections can affect viral swarm composition. As hosts, we have exploited the high mutation rates of viruses by redirecting viral evolution toward error catastrophe via pharmaceutical interventions [18, 19]. Interestingly, high-fidelity foot-and-mouth disease viral variants possess a higher level of resistance to pharmacologics but are attenuated in vivo, suggesting that the resulting restricted quasispecies hampers adaptability in the presence of drug or host pressures [19]. Also, antiviral treatment can lead genetic diversity gains that may precede selection of drug-resistant genotypes, as has been observed with oseltamivir [33, 35].

## Is there evidence for altered viral evolution in malnourished hosts?

Globally, 1 in 9 people are undernourished and 1 in 3 are overweight or obese, with innumerable others suffering from micronutrient deficiencies [1]. Consequently, it is of utmost importance to understand whether host nutrition actively shapes how viruses evolve because many hosts do not mirror the actively studied “wild-type” condition. Previous work has identified micronutrient deficiencies that may increase pathogen virulence through acquisition of minor variants. In mineral- and vitamin-deficient mice, genetic mutations arise in coxsackie B and influenza virus populations that promote virulence even in well-nourished hosts [36–40].

In our work with influenza virus, we determined that nutrient excesses can drive virulence through population diversification [41]. Experimental evolution of CA/09 virus through two models of murine obesity resulted in a viral population displaying increased virulence upon inoculation of a wild-type host. This phenotype was not strain specific; an avirulent H3N2 virus was, upon passage in obese hosts, able to productively infect immunocompetent mice. We observed a significant increase in viral diversity and subsequent virulence after a single round of infection, with the phenotype persisting in obese-derived viral populations across 10 passages [41]. Interestingly, arbovirus-infected obese or protein-deficient mice showed higher morbidity but lower viral diversity, and both malnourished models transmitted virus less efficiently, highlighting that the effects of nutrition may vary based on the natural life cycles of viral families [42]. It is yet to be determined how malnourishment may impact transmission of a respiratory, as compared with a vector-borne, virus.

## How could what we eat shape our viral pathogens?

Both undernourishment and obesity are two sides of the same coin and are implicated in blunting immune responses and increasing susceptibility to infection [43, 44]. In our studies with influenza virus, we linked the emergence of a more diverse and virulent viral population with blunted interferon responses in obese hosts. Interferon treatment of obese mice restricted the emergence of a diverse quasispecies and attenuated the virulence of the resulting viral population, strengthening the claim that a robust innate immune response restricts subsequent infection severity, possibly through reduced viral replication and acquisition of a genetically diverse viral population [8, 20, 41]. Dietary metabolites also influence cellular metabolism and can push the body to a state of metainflammation; this prooxidant environment may also directly influence the genetic composition of the viral population [45].

**Fig 2:**
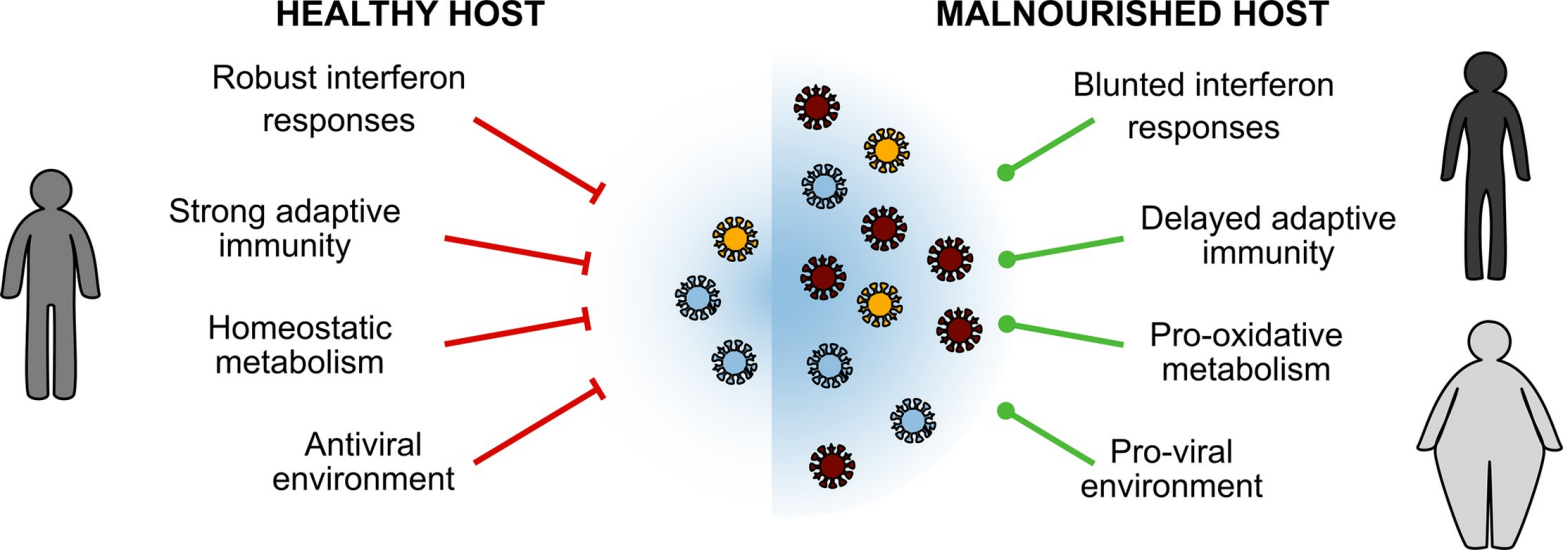
Direct effects of malnutrition on host antiviral responses shape the viral population. Nutritional status can alter the baseline state of host cells and the antiviral response postinfection. These departures to healthy metabolism and robust immunity may cultivate a proviral environment for increased replication and perhaps permit acquisition of beneficial mutations due to reduced selective pressures on the viral population. Other indirect effects of malnutrition could also alter viral interactions with resident microbiota. Finally, the ripple effects of over- and undernutrition are systemic for the host; however, nutrition status may have differing impacts for the virus based on tissue tropism, route of transmission, and mode of pathology.

Nutritional excess or deficiency may dampen the host immune responses and alter cellular metabolism, indirectly fostering an advantageous environment for viruses to explore the sequence space (**Fig 2**). The dearth of host responses to infection—particularly innate immunity—and the baseline malnourished state facilitates greater viral replication, permits the diversification of the viral swarm, and potentially allows for the emergence of advantageous mutations. Other indirect consequences of poor nutrition may also be involved. Blunting of immune responses may alter viral tropism and viral- or immune-induced pathology, thus remodeling the microenvironment in which the virus attacks the host. Also, nutrition is increasingly appreciated as an influence on the gut microbiome (reviewed in [46]). Interestingly, perturbations to the microbiome—both respiratory and gut—dampen interferon responses to respiratory virus infection [47–49]. However, to our knowledge, no empirical studies connect the obese microbiome to modulating enteric or respiratory viral populations.

## Conclusion

Pathogen virulence is a complex interplay of both host and pathogen properties. Host nutritional status has long been considered a risk for infection susceptibility and severity and is now implicated in shaping viral evolution. Continued studies on the molecular consequences of obesity and malnutrition at the macro- and micronutrient levels will reveal which host defenses are impaired through malnutrition and how they control quasispecies development and viral pathogenesis. Similarly, as we gain insight into how hosts influence quasispecies formation and pathogen virulence, we too can exploit these features for host benefit [18, 50].

The global ubiquity of malnutrition is shifting our population toward a more susceptible state. This will undoubtedly influence how pathogens behave within and between hosts. Continued study of how quasispecies evolution relates to other human, animal, and plant pathogens will indeed usher in a greater understanding of host–pathogen interactions and provide novel insights into how pathogens impact hosts and hosts impact pathogens.

## References

[1] Global Nutrition Report. 2020 Global Nutrition Report: Action on equity to end malnutrition. Bristol, UK: Development Initiatives, 2020.

[2] Epstein IR, Eigen M. Selection and self-organization of self-reproducing macromolecules under the constraint of constant flux. Biophys Chem. 1979;10(2):153–60. 10.1016/0301-4622(79)85035-8 . 486701

[3] Duarte EA, Novella IS, Ledesma S, Clarke DK, Moya A, Elena SF, et al Subclonal components of consensus fitness in an RNA virus clone. J Virol. 1994;68(7):4295–301. 10.1128/JVI.68.7.4295-4301.1994 8207804 PMC236352

[4] Kattenbelt JA, Stevens MP, Selleck PW, Gould AR. Analysis of Newcastle disease virus quasispecies and factors affecting the emergence of virulent virus. Arch Virol. 2010;155(10):1607–15. 10.1007/s00705-010-0739-4 20602243 PMC7086669

[5] Arenas JI, Gallegos-Orozco JF, Laskus T, Wilkinson J, Khatib A, Fasola C, et al Hepatitis C virus quasi-species dynamics predict progression of fibrosis after liver transplantation. J Infect Dis. 2004;189(11):2037–46. 10.1086/386338 . 15143471

[6] Domingo E, Holland JJ. RNA virus mutations and fitness for survival. Annu Rev Microbiol. 1997;51:151–78. 10.1146/annurev.micro.51.1.151 . 9343347

[7] Domingo E, Sheldon J, Perales C. Viral quasispecies evolution. Microbiology and molecular biology reviews: MMBR. 2012;76(2):159–216. 10.1128/MMBR.05023-11 22688811 PMC3372249

[8] Wasik BR, Muñoz-Rojas AR, Okamoto KW, Miller-Jensen K, Turner PE. Generalized Selection to Overcome Innate Immunity Selects for Host Breadth in an RNA Virus. Evolution; international journal of organic evolution. 2016;70(2). 10.1111/evo.12845 . 26882316

[9] Garcia-Arriaza J, Manrubia SC, Toja M, Domingo E, Escarmis C. Evolutionary transition toward defective RNAs that are infectious by complementation. Journal of Virology. 2004;78(21):11678–85. 10.1128/JVI.78.21.11678-11685.2004 WOS:000224540900022. 15479809 PMC523252

[10] Vignuzzi M, Stone JK, Arnold JJ, Cameron CE, Andino R. Quasispecies diversity determines pathogenesis through cooperative interactions in a viral population. Nature. 2006;439(7074):344–8. 10.1038/nature04388 16327776 PMC1569948

[11] Koelle K, Rasmussen DA. The effects of a deleterious mutation load on patterns of influenza A/H3N2's antigenic evolution in humans. eLife. 2015;4:e07361 10.7554/eLife.07361 26371556 PMC4611170

[12] Sanz-Ramos M, Diaz-San Segundo F, Escarmis C, Domingo E, Sevilla N. Hidden virulence determinants in a viral quasispecies in vivo. J Virol. 2008;82(21):10465–76. 10.1128/JVI.00825-08 18715925 PMC2573215

[13] Pfeiffer JK, Kirkegaard K. Bottleneck-mediated quasispecies restriction during spread of an RNA virus from inoculation site to brain. Proc Natl Acad Sci U S A. 2006;103(14):5520–5. 10.1073/pnas.0600834103 16567621 PMC1414638

[14] Domingo E, Sabo D, Taniguchi T, Weissmann C. Nucleotide sequence heterogeneity of an RNA phage population. Cell. 1978;13(4):735–44. Epub 1978/04/01. 10.1016/0092-8674(78)90223-4 . 657273

[15] Braun T, Borderia AV, Barbezange C, Vignuzzi M, Louzoun Y. Long-term context-dependent genetic adaptation of the viral genetic cloud. Bioinformatics. 2019;35(11):1907–15. 10.1093/bioinformatics/bty891 . 30346482

[16] Delgado-Eckert E, Ojosnegros S, Beerenwinkel N. The evolution of virulence in RNA viruses under a competition-colonization trade-off. Bull Math Biol. 2011;73(8):1881–908. 10.1007/s11538-010-9596-2 . 21082274

[17] Lancaster KZ, Pfeiffer JK. Viral population dynamics and virulence thresholds. Current opinion in microbiology. 2012;15(4):525–30. 10.1016/j.mib.2012.05.007 22658738 PMC3424342

[18] Ruiz-Jarabo CM, Ly C, Domingo E, de la Torre JC. Lethal mutagenesis of the prototypic arenavirus lymphocytic choriomeningitis virus (LCMV). Virology. 2003;308(1):37–47. 10.1016/s0042-6822(02)00046-6 . 12706088

[19] Zeng J, Wang H, Xie X, Li C, Zhou G, Yang D, et al Ribavirin-resistant variants of foot-and-mouth disease virus: the effect of restricted quasispecies diversity on viral virulence. J Virol. 2014;88(8):4008–20. 10.1128/JVI.03594-13 24453363 PMC3993757

[20] Fitzsimmons WJ, Woods RJ, McCrone JT, Woodman A, Arnold JJ, Yennawar M, et al A speed-fidelity trade-off determines the mutation rate and virulence of an RNA virus. PLoS Biol. 2018;16(6):e2006459 10.1371/journal.pbio.2006459 29953453 PMC6040757

[21] Pfeiffer JK, Kirkegaard K. Increased fidelity reduces poliovirus fitness and virulence under selective pressure in mice. PLoS Pathog. 2005;1(2):e11 10.1371/journal.ppat.0010011 16220146 PMC1250929

[22] Holmes EC, Moya A. Is the quasispecies concept relevant to RNA viruses? J Virol. 2002;76(1):460–5. 10.1128/jvi.76.1.460-462.2002 11739715 PMC135735

[23] Topfer A, Hoper D, Blome S, Beer M, Beerenwinkel N, Ruggli N, et al Sequencing approach to analyze the role of quasispecies for classical swine fever. Virology. 2013;438(1):14–9. 10.1016/j.virol.2012.11.020 . 23415390

[24] Jenckel M, Blome S, Beer M, Hoper D. Quasispecies composition and diversity do not reveal any predictors for chronic classical swine fever virus infection. Arch Virol. 2017;162(3):775–86. 10.1007/s00705-016-3161-8 . 27885563

[25] Farci P, Shimoda A, Coiana A, Diaz G, Peddis G, Melpolder JC, et al The outcome of acute hepatitis C predicted by the evolution of the viral quasispecies. Science. 2000;288(5464):339–44. 10.1126/science.288.5464.339 . 10764648

[26] Honda M, Kaneko S, Sakai A, Unoura M, Murakami S, Kobayashi K. Degree of diversity of hepatitis C virus quasispecies and progression of liver disease. Hepatology. 1994;20(5):1144–51. 10.1002/hep.1840200507 . 7927245

[27] Korboukh VK, Lee CA, Acevedo A, Vignuzzi M, Xiao Y, Arnold JJ, et al RNA virus population diversity, an optimum for maximal fitness and virulence. The Journal of biological chemistry. 2014;289(43):29531–44. 10.1074/jbc.M114.592303 25213864 PMC4207971

[28] Parameswaran P, Wang C, Trivedi SB, Eswarappa M, Montoya M, Balmaseda A, et al Intrahost Selection Pressures Drive Rapid Dengue Virus Microevolution in Acute Human Infections. Cell Host Microbe. 2017;22(3):400–10 e5. 10.1016/j.chom.2017.08.003 28910637 PMC5616187

[29] Vega E, Donaldson E, Huynh J, Barclay L, Lopman B, Baric R, et al RNA populations in immunocompromised patients as reservoirs for novel norovirus variants. J Virol. 2014;88(24):14184–96. 10.1128/JVI.02494-14 25275120 PMC4249157

[30] Chaudhry A, Bastien N, Li Y, Scott A, Pabbaraju K, Stewart D, et al Oseltamivir resistance in an influenza A (H3N2) virus isolated from an immunocompromised patient during the 2014–2015 influenza season in Alberta, Canada. Influenza and other respiratory viruses. 2016;10(6):532–5. 10.1111/irv.12415 27442795 PMC5059956

[31] Kleine-Weber H, Elzayat MT, Wang L, Graham BS, Muller MA, Drosten C, et al Mutations in the Spike Protein of Middle East Respiratory Syndrome Coronavirus Transmitted in Korea Increase Resistance to Antibody-Mediated Neutralization. J Virol. 2019;93(2). 10.1128/JVI.01381-18 30404801 PMC6321919

[32] Gay RT, Belisle S, Beck MA, Meydani SN. An aged host promotes the evolution of avirulent coxsackievirus into a virulent strain. Proc Natl Acad Sci U S A. 2006;103(37):13825–30. 10.1073/pnas.0605507103 16950876 PMC1564236

[33] Roosenhoff R, van der Vries E, van der Linden A, van Amerongen G, Stittelaar KJ, Smits SL, et al Influenza A/H3N2 virus infection in immunocompromised ferrets and emergence of antiviral resistance. PLoS ONE. 2018;13(7):e0200849 10.1371/journal.pone.0200849 ; PubMed Central PMCID:30024940 PMC6053203

[34] van der Vries E, Stittelaar KJ, van Amerongen G, Veldhuis Kroeze EJB, de Waal L, Fraaij PLA, et al Prolonged Influenza Virus Shedding and Emergence of Antiviral Resistance in Immunocompromised Patients and Ferrets. PLoS Pathog. 2013;9(5);e1003343 10.1371/journal.ppat.1003343 23717200 PMC3662664

[35] Mori K, Murano K, Ohniwa RL, Kawaguchi A, Nagata K. Oseltamivir expands quasispecies of influenza virus through cell-to-cell transmission. Scientific reports. 2015;5:9163 10.1038/srep09163 25772381 PMC4649863

[36] Beck MA, Shi Q, Morris VC, Levander OA. Rapid genomic evolution of a non-virulent coxsackievirus B3 in selenium-deficient mice results in selection of identical virulent isolates. Nature medicine. 1995;1(5):433–6. 10.1038/nm0595-433 . 7585090

[37] Beck MA, Nelson HK, Shi Q, Van Dael P, Schiffrin EJ, Blum S, et al Selenium deficiency increases the pathology of an influenza virus infection. FASEB J. 2001;15(8):1481–3. 10.1096/fj.00-0721fje . 11387264

[38] Nelson HK, Shi Q, Van Dael P, Schiffrin EJ, Blum S, Barclay D, et al Host nutritional selenium status as a driving force for influenza virus mutations. FASEB J. 2001;15(10):1846–8. . 11481250

[39] Beck MA, Shi Q, Morris VC, Levander OA. From avirulent to virulent: Vitamin E deficiency in mice drives rapid genomic evolution of a coxsackffi B3 virus. 1996;10(3).

[40] Beck MA, Handy J, Levander OA. Host nutritional status: the neglected virulence factor. Trends in microbiology. 2004;12(9):417–23. 10.1016/j.tim.2004.07.007 15337163 PMC7127785

[41] Honce R, Karlsson EA, Wohlgemuth N, Estrada LD, Meliopoulos VA, Yao J, et al Obesity-Related Microenvironment Promotes Emergence of Virulent Influenza Virus Strains. mBio. 2020;11(2). 10.1128/mBio.03341-19 32127459 PMC7064783

[42] Weger-Lucarelli J, Carrau L, Levi LI, Rezelj V, Vallet T, Blanc H, et al Host nutritional status affects alphavirus virulence, transmission, and evolution. PLoS Pathog. 2019;15(11):e1008089 10.1371/journal.ppat.1008089 31710653 PMC6872174

[43] Taylor AK, Cao W, Vora KP, De La Cruz J, Shieh WJ, Zaki SR, et al Protein energy malnutrition decreases immunity and increases susceptibility to influenza infection in mice. J Infect Dis. 2013;207(3):501–10. 10.1093/infdis/jis527 . 22949306 PMC11341849

[44] Flanigan CC, Sprunt DH. The effect of malnutrition on the susceptibility of the host to viral infection. The Journal of experimental medicine. 1956;104(5):687–706. 10.1084/jem.104.5.687 13367338 PMC2136615

[45] Smith AD, Botero S, Levander OA. Copper deficiency increases the virulence of amyocarditic and myocarditic strains of coxsackievirus B3 in mice. J Nutr. 2008;138(5):849–55. 10.1093/jn/138.5.849 . 18424590

[46] Maruvada P, Leone V, Kaplan LM, Chang EB. The Human Microbiome and Obesity: Moving beyond Associations. Cell Host Microbe. 2017;22(5):589–99. 10.1016/j.chom.2017.10.005 . 29120742

[47] Steed AL, Christophi GP, Kaiko GE, Sun L, Goodwin VM, Jain U, et al The microbial metabolite desaminotyrosine protects from influenza through type I interferon. Science. 2017;357(6350):498–502. 10.1126/science.aam5336 28774928 PMC5753406

[48] Tomosada Y, Chiba E, Zelaya H, Takahashi T, Tsukida K, Kitazawa H, et al Nasally administered Lactobacillus rhamnosus strains differentially modulate respiratory antiviral immune responses and induce protection against respiratory syncytial virus infection. BMC Immunol. 2013;14:40 10.1186/1471-2172-14-40 23947615 PMC3751766

[49] Ichinohe T, Pang IK, Kumamoto Y, Peaper DR, Ho JH, Murray TS, et al Microbiota regulates immune defense against respiratory tract influenza A virus infection. Proc Natl Acad Sci U S A. 2011;108(13):5354–9. 10.1073/pnas.1019378108 21402903 PMC3069176

[50] Moratorio G, Henningsson R, Barbezange C, Carrau L, Borderia AV, Blanc H, et al Attenuation of RNA viruses by redirecting their evolution in sequence space. Nat Microbiol. 2017;2:17088 10.1038/nmicrobiol.2017.88 28581455 PMC7098180

